# Diagnostic utility of alarm features in predicting malignancy in patients with dyspeptic symptoms

**DOI:** 10.1007/s12664-021-01155-x

**Published:** 2021-04-08

**Authors:** Anurag Shetty, Girisha Balaraju, Shiran Shetty, Cannanore Ganesh Pai

**Affiliations:** 1 Department of Gastroenterology and Hepatology, Kasturba Medical College, Mangalore, Manipal Academy of Higher Education, Manipal, 575 003 India; 2 Department of Gastroenterology and Hepatology, Kasturba Medical College, Manipal, Manipal Academy of Higher Education, Manipal, 576 104 India

**Keywords:** Abdominal pain, Alarm symptoms, Diagnostic accuracy, Dyspepsia, Endoscopy, Evaluation, India, Management, Risk factors, Surveillance, Upper gastrointestinal malignancy

## Abstract

**Background:**

Clinical features are of modest benefit in determining the etiology of dyspepsia. Dyspeptic patients with alarm features are suspected to have malignancy; but the proportions of patients and true cutoff values of various quantitative parameters in predicting malignancy are explored to a lesser extent.

**Methods:**

This is a prospective observational study of consecutive patients undergoing esophagogastroduodenoscopy (EGD) for dyspeptic symptoms. Patients’ alarm features and clinical details were recorded in a predesigned questionnaire. The diagnostic accuracy of alarm features in predicting malignancy was studied.

**Results:**

Nine hundred patients, 678 (75.3%) males, with a mean (standard deviation [SD]) age of 44.6 (13.54) years were enrolled. Commonest indication for EGD was epigastric pain in 614 (68.2%) patients. Dyspepsia was functional in 311 (34.6%) patients. EGD revealed benign lesions in 340 (37.8%) and malignancy in 50 (5.5%) patients. Among the malignant lesions, gastric malignancy was present in 28 (56%) and esophageal malignancy in 20 (40%) patients. Alarm features were present in 206 (22.9%), out of which malignant lesions were seen in 46 (22.3%) patients. Altogether, the alarm features had a sensitivity of 92% and specificity of 81.2% for predicting malignancy. The sensitivity and specificity for weight loss were 76% and 90.8%, while that of abdominal mass were 10% and 99.9% respectively. Based on receiver operating characteristic curve, the optimal age for screening of malignancy was 46.5 years in this population.

**Conclusions:**

Patients of age group 40 to 49 years with dyspeptic alarm symptoms (predominant weight loss) need prompt endoscopy to screen for malignancy. The alarm features are inexpensive screening tools, found to be useful in India, and should be utilized in countries with similar healthcare conditions and disease epidemiology.

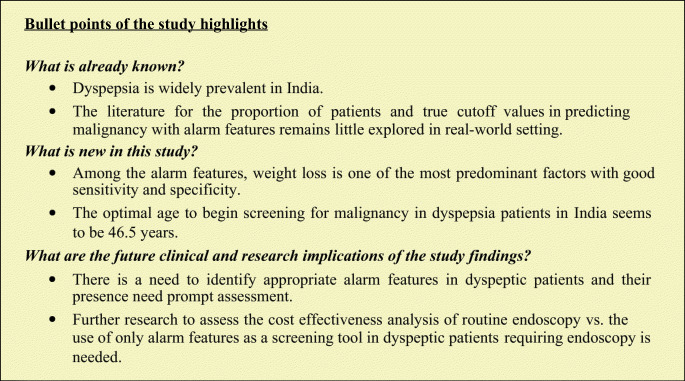



## Introduction

Dyspepsia presents with persistent or recurrent pain or discomfort in the upper abdomen in addition to other gastrointestinal (GI) symptoms [1]. It is the commonest condition seen by gastroenterologists on a daily basis in which organic causes are seen only in a few [2]. Its prevalence varies from 25% to 40% in western countries [3] while the prevalence in Asian population is 8% to 30% [4]. It leads to considerable impact on treatment as the estimated direct annual cost incurred in treating dyspepsia is over $12 billion in the USA [5]. Multiple conditions may mimic dyspepsia or may underlie dyspepsia, including gastroesophageal reflux disease (GERD), peptic ulcer, and most importantly GI malignancy, which contribute to 1% to 3% of all patients with dyspepsia [6,7]. In more than 50% of the patients with uninvestigated dyspepsia, nor obvious organic cause is found (a condition called functional dyspepsia) [8]. Esophagogastroduodenoscopy (EGD) helps in the initial diagnosis but numerous attempts at recognizing patients most likely to benefit from endoscopic evaluation have not been successful. The salient feature of any empirical treatment for dyspepsia is to minimize the risk of missing a GI malignancy at a treatable stage. Alarm features and age were of limited value for predicting malignancy in patients with dyspepsia from Asian countries [9]. Dyspeptic patients with alarm features are suspected to have malignancy; but the proportion of patients and true cutoff values of various quantitative parameters in predicting malignancy are explored to a lesser extent in Asian countries. The diagnostic utility of dyspeptic alarm symptoms in predicting who has malignancy is unclear [9]. So, this study is aimed to determine the spectrum of malignancies in patients with dyspepsia and frequency of alarm features and to evaluate the diagnostic accuracy of these alarm features in predicting upper GI malignancy.

## Methods

This is a prospective observational study of consecutive patients undergoing EGD for dyspeptic symptoms at a tertiary care teaching hospital from November 2013 to December 2014. The study protocol was approved by the Institutional Ethics Committee (Approval Number IEC 547/2013). A written informed consent was obtained from all the patients prior to their enrolment. This study was conducted in accordance with Good Clinical Practice and in a manner to conform to the Helsinki Declaration of 1975, as revised in 2000 and 2008 concerning human rights. The inclusion criteria was dyspepsia of greater than 4 weeks (ROME III criteria) in adult patients (>18 year) [10]. The exclusion criteria were pregnancy, cholelithiasis, pancreatitis, history of surgery of the esophagus or stomach, previously diagnosed cirrhosis of the liver, treated malignancy, use of proton pump inhibitors daily for >4 weeks duration, predominant heartburn, anemia weight loss, predominant dysphagia, and refusal of consent. Relevant clinical details regarding concomitant medication, addictions, height, weight, body mass index, and alarm features like anemia, significant weight loss defined as unintended weight loss of more than 10% over 3 months, abdominal mass palpable, supraclavicular lymph nodes, persistent vomiting, GI bleeding, dysphagia, and family history of malignancies were recorded in a predesigned questionnaire.

Standard EGD (GIF-N180 [Olympus, Tokyo, Japan]) was performed by experienced gastroenterologists in all the patients under local anesthesia and endoscopic findings were recorded. In case of suspected malignancy, multiple biopsy specimens were taken from the suspected lesion and sent for histological confirmation. The biopsy specimen was interpreted by an expert pathologist. Treatment for dyspepsia was done as per appropriate guidelines [11].

### Statistical analysis

The Statistical Package for Social Sciences version 16 (SPSS Inc., Chicago, IL, USA) for windows was used for the statistical analysis. Descriptive statistics were used as appropriate. The Chi-square test was used for categorical data, while analysis of variance (ANOVA) and Student’s *t* test were used for continuous data. The diagnostic values of individual alarm feature, including sensitivity, specificity, positive predictive value (PPV), and negative predictive value (NPV), were calculated. Based on this, area under the receiver operating characteristic (ROC) curve was used to find out an optimal age for screening of malignancy. A *p*-value of <0.05 was considered to be statistically significant.

## Results

### Study population

Nine hundred patients, 678 (75.3%) males, with a mean (SD) age of 44.6 (13.54) years with majority (648 [72%]) being in the age group of 25–55 years were included. Hypertension was the most prevalent comorbidity seen in 89 (9.9%) patients (Table 1). The commonest indication for EGD was epigastric pain in 614 (68.2%) patients. Dyspepsia was functional in 311 (34.6%) patients. EGD revealed benign lesions in 340 (37.8%) and malignancy in 50 (5.5%) patients. Among the histologically confirmed malignant lesions, gastric malignancy was present in 28 (56%), esophageal malignancy in 20 (40%), and duodenal malignancy in 2 (8%) patients. On analyzing the relationship of duration of dyspepsia with malignancy, it was found that the patients with malignancy had a mean (SD) duration of dyspepsia of 5.14 (16.84) months compared to 26.8 (45.7%) in patients without malignancy which was statistically significant (*p* < 0.001). Patients with lower hemoglobin levels had higher proportion of malignancy. 13/61 (21.3%) patients with hemoglobin levels <10 g/dL had malignancy, whereas only 16/571 (2.8%) with hemoglobin levels >13 g/dL had malignancy (*p* < 0.01). Alcohol in 20 (40%) and smoking in 19 (38%) patients were found to have an association with malignancy (*p* < 0.05).

**Table 1:** Baseline characteristics of patients

	*Number* (%)
Parameter	
Total number of patients	900 (100)
Age, mean (SD) in year	44.6 (13.54)
Symptom duration in months, median (range)	8 (1–360)
Gender, M:F	678 (75.3), 3.05:1
BMI (kg/m^2^), mean (SD)	24.5 (4.2)
Smoking	152 (16.9)
Alcoholism	219 (24.3)
Tobacco use	106 (11.8)
Comorbid conditions	
Hypertension	89 (9.9)
Diabetes mellitus	54 (6)
Ischemic heart disease	22 (2.4)
Bronchial asthma/COPD	14 (1.5)
Cerebrovascular accident	7 (0.8)
Presenting alarm features^#^	
Weight loss	116 (12.9)
Dysphagia	52 (5.8)
Persistent vomiting	40 (4.4)
Gastrointestinal bleeding	44 (4.9)
Abdominal mass	6 (0.7)
Family history of malignancy	13 (1.4)
Anemia	79 (8.9)
Supraclavicular lymph nodes	4 (0.4)
Indication for EGD in addition to alarm features^#^	
Epigastric pain	614 (68.2)
Epigastric burning	569 (63.2)

^#^Some patients may have more than one presenting /alarm symptoms

*SD* standard deviation, *M* male, *F* female, *BMI* body mass index, *COPD* chronic obstructive pulmonary disease, *EGD* esophagogastroduodenoscopy

### Characterization of alarm features

**Fig. 1:**
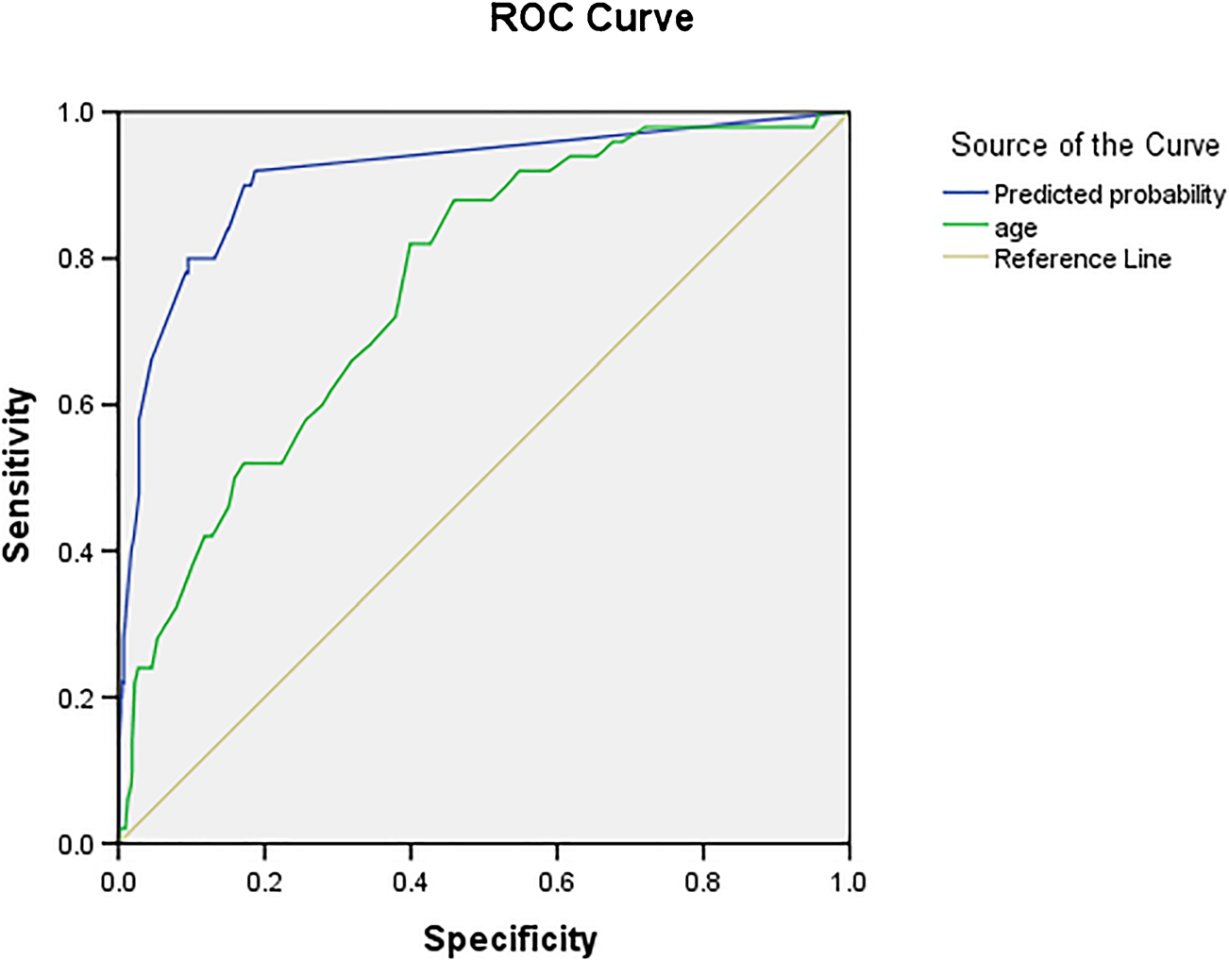
Receiver operating characteristic curve for age and malignancy

Alarm features were present in 206 (22.9%), out of whom malignant lesions were seen in 46 (22.3%) patients (Table 2). Weight loss was the most common alarm feature reported in 116 (12.9%) patients. The PPV, NPV, sensitivity, and specificity of finding a malignancy in patients with various alarm features are given in Table 3. Altogether, the alarm features had a sensitivity of 92% and specificity of 81.2% for predicting malignancy. All the alarm features put together had a high NPV of 99.4% with each alarm feature having a NPV beyond 95%. The sensitivity and specificity for weight loss were 76% and 90.8%, while that of palpable abdominal mass was 10% and 99.9% respectively. Abdominal mass and palpable supraclavicular lymph nodes showed the highest probability of predicting malignancy (83.3% [5/6] and 50% [2/4] respectively). Also the PPV of the alarm features put together increased with increasing age from 8.2% in the age group of 18–40 reaching 50% in those in the age group of 80–100. Based on the ROC curve (Fig. 1), the optimal age for screening of malignancy was 46.5 years with a ROC of 0.763 (fair), sensitivity of 82%, and specificity of 60%. When the age was combined with alarm features, the ROC of 0.914 (excellent) was obtained.

**Table 2:** Frequency of alarm features in dyspeptic patients with or without malignancy

Alarm features	Dyspepsia with malignancy, *n* = 50, *n* (%)^#^	Dyspepsia without malignancy, *n* = 850, *n* (%)^#^
Any alarm feature	46 (92)	160 (18.8)
Weight loss	38 (76)	78 (9.1)
Dysphagia	19 (38)	33 (3.8)
Anemia	19 (38)	60 (7)
Persistent vomiting	12 (24)	28 (3.3)
Gastrointestinal bleed	7 (14)	37 (4.3)
Abdominal mass	5 (10)	1 (0.1)
Family history of malignancy	5 (10)	8 (0.9)
Supraclavicular lymph node	2 (4)	2 (0.2)

^#^Some patients may have more than one alarm feature

**Table 3:** Positive predictive value, negative predictive value, sensitivity, and specificity of alarm features

Alarm features	Positive predictive value (%)	Negative predictive value (%)	Sensitivity (%)	Specificity (%)
Any alarm feature	22.3	99.4	92	81.2
Weight loss	32.7	98.4	76	90.8
Dysphagia	36.5	96.3	38	96.1
Persistent vomiting	30	95.5	24	96.7
Gastrointestinal bleed	15.9	95	14	95.6
Abdominal mass	83.3	94.9	10	99.9
Family history of malignancy	38.5	94.9	10	99.1
Anemia	24	96.2	38	92.9
Supraclavicular lymph node	50	94.6	4	99.8

## Discussion

By prospectively analyzing patients with dyspepsia over a 14-month period, we found that alarm features are effective tools to identify malignant lesions in these patients. Malignancy was seen in 5.5% of our patients, while the reported frequency of malignancy in patients with dyspepsia from India varies from 3.9% to 8.3% [12,13], In our study, gastric malignancies accounted for 56%, which was somewhat similar to studies conducted across the country, wherein malignant pathology was more often a gastric malignancy [12,13].

Weight loss was the most common alarm feature seen in 12.9% of our patients followed by dysphagia (5.8%), while palpable supraclavicular lymph nodes (0.4%) was the least common. However, Indian data have reported dysphagia as the most frequent alarm feature seen in up to 10% patients [12, 13]. This variation could be attributed to the fact that patients presenting with predominant dysphagia without associated dyspepsia were not part of our study. In patients with alarm symptoms, malignant lesions were found in 22.3%, which was comparable to other studies with a reported frequency of 25% [12]. Abdominal mass and palpable supraclavicular lymph nodes showed the highest probability of finding a malignancy of 83.3% and 50% respectively. The NPV of alarm features altogether was 99.4% with each of the individual alarm features having NPV of 95% or greater similar to that seen in another study [14], which makes it very attractive with little chance of missing an underlying malignancy.

A few meta-analyses described significant heterogeneity between the studies [15, 16]. The sensitivity and specificity of alarm symptoms varied significantly from 0% to 83% and 40% to 98% respectively [15]. The presence of any alarm feature in our study showed a sensitivity of 92% and a specificity of 81.2%. Among individual alarm features, weight loss had the highest sensitivity of 76% with a specificity of 90.8%. Anemia and dysphagia had a sensitivity of 38% each. The rest of the alarm features had a very low sensitivity of <25% each of which was almost similar to the data in the meta-analysis by Fransen et al. [16]. There is an increase in the risk of gastric and esophageal cancer with advancing age, but a cutoff age is currently unavailable [17]. The age recommendation for endoscopy in patients with dyspepsia should be decided partly by the incidence of malignancy in that country. The American Gastroenterological Association’s position statement recommends a cutoff age of 55 years for endoscopy [18]. Whereas, the Asian guidelines recommend endoscopy in new-onset dyspeptic patients over 40 years of age in areas of high prevalence and over 45 and 50 years in areas of intermediate and low prevalence respectively. Bangladesh, India, and Thailand are considered low-risk countries [19]. The optimal age for malignancy screening was estimated to be 46.5 years in our study with a sensitivity of 82% and specificity of 60% with an area under curve (AUC) of 0.763 similar to that reported by Khademi et al. [20] which is far below the cutoff age recommended by Asian or western guidelines. The AUC increased to 0.914 in our study when age was combined with the other alarm features.

We found that dyspeptic patients with underlying malignancy had a shorter mean duration of symptoms (< 6 months) when compared with those who did not harbor malignancy, This was statistically significant (*p* < 0.001; see the results section). Higher proportion of dyspeptic patients with hemoglobin levels <10 g/dL (21.3%) showed presence of malignancies compared with those with hemoglobin levels >10 g/dL (4.4%) (*p* < 0.001), making it an ideal hemoglobin level to begin screening for malignancies. The alarm features put together had a very high NPV (99.4%) which makes the chances of missing of malignancy was less likely, i.e., 1:166 chance of missing malignancy in those without alarm features. The present study had a few limitations. This was a single-centered study, with noncomparative design done in a tertiary care referral center; the data may not be representative of the community in general. There may be an element of referral bias. In ideal scenarios, each community should derive their own cutoff age from well-conducted local community studies.

To summarize, alarm features are effective and inexpensive tools to identify malignant lesions in patients with dyspepsia. Patients of age group 40 to 49 years with dyspeptic alarm symptoms (predominantly weight loss) need prompt endoscopy to screen for malignancy. Routine endoscopic surveillance should be limited to patients with alarm features. Alarm symptoms are useful in India and these results are applicable to countries with similar epidemiologic, socioeconomic, and healthcare conditions.
